# THE EFFECTS OF HEARING LOSS AND MOBILITY DECLINE ON COGNITIVE FUNCTION OF OLDER ADULTS

**DOI:** 10.1093/geroni/igz038.3387

**Published:** 2019-11-08

**Authors:** Ryota Sakurai, Hisashi Kawai, Shuich Yanai, Hunkyung Kim, Hirohiko Hirano, Kazushige Ihara, Shuichi Obuchi, Yoshinori Fujiwara

**Affiliations:** 1 Tokyo Metropolitan Institute of Gerontology, Itabashi, Tokyo, Japan; 2 Tokyo Metropolitan Institute of Gerontology, Tokyo, Japan; 3 Hirosaki University Graduate School of Medicine, Aomori, Japan

## Abstract

Background: Both hearing loss and mobility decline are well-known risk factors of cognitive impairment among older adults. However, the effects of the accumulation of these functional impairments are still unclear. Thus, the present study examined whether the interactive effects of hearing loss and poor gait performance contribute to cognitive impairments. Methods: Hearing loss and gait performance were assessed in 716 community-dwelling older adults at baseline. Pure-tone audiometry was conducted to determine hearing loss at 1 (i.e., low tone) and 4 kHz (i.e., high tone). Poor gait performance was defined as the lowest quartile (fourth quartile) of age- and sex-appropriate mean gait velocity. Participants were then classified into four groups according to the presence of hearing loss and poor gait performance. Cognitive function was assessed using MMSE and MoCA at baseline and four years later. Results: Older adults who had either hearing loss (low or high tone) or poor gait performance showed lower MMSE and MoCA scores at baseline. Multiple regression models showed that hearing loss and poor gait performance at baseline were significantly associated with decreased cognitive function at follow-up. Among older adults with low tone hearing loss, absence of slow gait was not associated with decreased cognitive function. Conclusions: Our results indicate the possibility that hearing loss and slow gait synergistically increase the risk of cognitive impairment. The results also suggest that the effect of slow gait on cognition exceeds the effects of hearing loss, indicating the importance of maintaining mobility in late life.

